# Selective Phosphodiesterase 4B Inhibitors: A Review

**DOI:** 10.3797/scipharm.1404-08

**Published:** 2014-06-10

**Authors:** Mohammed Afzal Azam, Naga Srinivas Tripuraneni

**Affiliations:** Department of Pharmaceutical Chemistry, J. S. S. College of Pharmacy, Ootacamund-643001, Tamil Nadu, India.

**Keywords:** Phosphodiesterases (PDE) enzymes, Cyclic adenosine monophosphate, Selective PDE inhibitors, Chronic obstructive pulmonary disease, Antiproliferative activity, PDE4B

## Abstract

Phosphodiesterase 4B (PDE4B) is a member of the phosphodiesterase family of proteins that plays a critical role in regulating intracellular levels of cyclic adenosine monophosphate (cAMP) by controlling its rate of degradation. It has been demonstrated that this isoform is involved in the orchestra of events which includes inflammation, schizophrenia, cancers, chronic obstructive pulmonary disease, contractility of the myocardium, and psoriatic arthritis. Phosphodiesterase 4B has constituted an interesting target for drug development. In recent years, a number of PDE4B inhibitors have been developed for their use as therapeutic agents. In this review, an up-to-date status of the inhibitors investigated for the inhibition of PDE4B has been given so that this rich source of structural information of presently known PDE4B inhibitors could be helpful in generating a selective and potent inhibitor of PDE4B.

## Introduction

Phosphodiesterases (PDEs) are a diverse family of enzymes involved in phosphoric diester hydrolytic cleavage. The term, however, is usually applied to phosphodiesterases that cleave cyclic nucleotides that are important for transmitting signals within the cell. These enzymes are known as cyclic nucleotide phosphodiesterases (PDEs). There are other families of phosphodiesterases, including phospholipases C and D, autotaxin, sphingomyelin phosphodiesterase, DNases, RNases, and restriction endonucleases. Cyclic nucleotide PDEs constitute a superfamily of enzymes which catalyze the hydrolysis of 3',5'-cyclic adenosine monophosphate (cAMP) and 3',5'-cyclic guanosine monophosphate (cGMP) to their inactive 5′-AMP and 5’-GMP forms, respectively. In mammals, PDE enzymes are classified into 11 families, namely PDE1-PDE11. PDE4 is differentiated from other PDEs by its high sensitivity towards the inhibitors, and because of its potential use in the treatment of depression and schizophrenia [1, 2].

PDE4 is the major cAMP-metabolizing enzyme found in inflammatory and immune cells [3, 4]. The PDE4 family encompasses four genes coding the subtypes PDE4A*,* PDE4B*,* PDE4C, and PDE4D [5]*,* but the PDE4B subtype is believed to play a central role in inflammation [6], being the predominant subtype in monocytes and neutrophils. All four PDE4 subtypes comprise a related structural organization with a highly conserved catalytic domain in the C-terminal region and upstream-conserved regions in the N-terminal portion of the protein [5]. The high-resolution structures of PDE4B complexes are presented and provide an insight into understanding the substrate and inhibitor binding as well as the critical function of metal ions [7, 8]. PDE4B comprises three domains: an N-terminal regulatory domain, a catalytic domain of about 300 amino acids, and a C-terminal domain. The catalytic domain is the most conserved domain among the PDE families and consists of 17 α-helices. Several reviews on PDE4 have appeared in articles [9–16] covering the role of phosphodiesterase-4 inhibitors in the treatment of asthma, chronic obstructive pulmonary, psoriasis, psoriatic arthritis, chronic inflammatory, autoimmune, and inflammatory bowel diseases. In this review, we present the most significant examples of PDE4B inhibitors that exhibit various biological activities reported in literature.

## Selective PDE4B Inhibitors

The design of selective PDE inhibitors started in 1958 when Sutherland and Rall [17] identified the enzymatic activity of phosphodiesterases and its biochemical importance. These cyclic nucleotide levels play an important role in many mammalian physiologies ranging from immune and inflammatory responses [18], regulation of the contractility of the myocardium and smooth muscles [19], to depression and cognition [20]. These centric roles of cAMP initiated research efforts in developing selective inhibitors of PDEs such as vinpocetin for PDE1, inamrinone, milrinone, enaximone for PDE2, rolipram, cilomilast, roflumilast for PDE4, and sildenafil for PDE5 [21]. A pharmacological study in mice proved that PDE4B mediates antipsychotic effects [22, 23] by its role in dopamine-associated and stress-related processes. PDE4B is the only subtype of PDE4 expressed in the locus coeruleus, a region in the brain that is rich in nonadrenergic neurons that mediates some anti-depressant effects [24] and it is the only isoform of PDE4 expressed in the white matter of the brain. PDE4B is also involved in schizophrenia [25] and anxiety [26]. In contrast to PDE4B, PDE4D is expressed in the area postrema and nucleus of the solitary tract [27, 28], which are responsible for emesis.

**Fig. 1.**
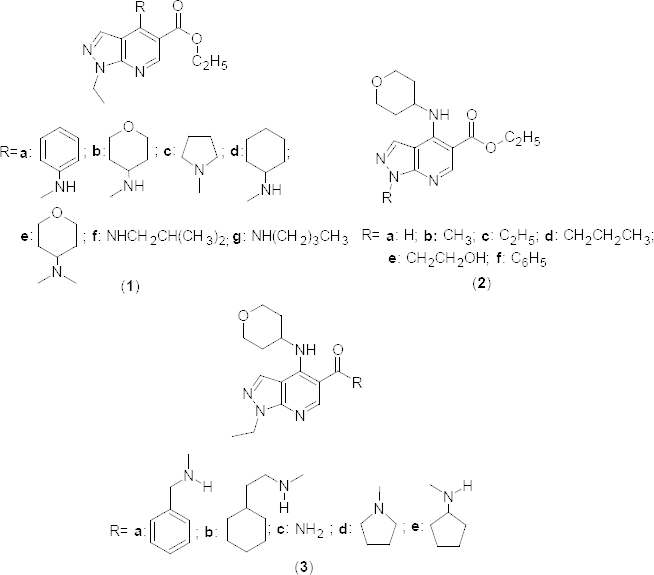
Structure of various congeners of pyrazolopyridine with PDE4B inhibitory activity

The first generation the PDE4 inhibitor rolipram was withdrawn for its potent action on the PDE4D isoform, which is likely to be implicated in emesis [30]. PDE4B is abundant in inflammatory, immune, and airway smooth muscle cells [31] and thoroughly investigated for treating inflammatory pulmonary disorders [11]. Further, gene knockout studies proved that PDE4B could suppress TNF-α production [32] and has been investigated as an attractive and excellent therapeutic target due to its sensitivity to selective inhibitors [33–35]. In the year 2004, a high throughput screening resulted in the identification of the lead compound **1a** (Figure 1), which exhibited significant PDE4B inhibition (pIC_50_6.8) [36]. A high temperature reaction between N-1-ethyl-5-aminopyrazole and diethyl ethoxymethylenemalonate resulted in the formation of the intermediate 4-hydroxypyrazolopyridine, which upon treatment with phosphoryl chloride gave the 4-chloro-derivative. Treatment of the 4-chloro-derivative with a diverse range of amines yielded **2a–g** (Figure 1) *via* thermal displacement [37]. Structure-activity data showed a significant increase in the potency with a branched or cycloalkyl amino group at fourth position of the pyridine nucleus, with 6-membered saturated rings being more potent than 5-membered rings. Tertiary amines such as pyrrolidine in **1c** and the N-methylated analogue such as **1e** were less tolerated and suggested the importance of the NH group. These observations were further supported by crystal structures of the pyrazolopyridine analogue **3a** (Figure 1) bound to PDE4B 152-503 (PDB ID: 3D3P), which showed the tetrahydropyran moiety towards the metal ions. An intramolecular hydrogen bond between the 4-amino NH group and the carbonyl of the amide was observed, which maintained the co-planarity with the template. Unbranched alkyl amines gave poor selectivity in comparison to the branched analogues, which routinely gave >100-fold selectivity.

The structure-activity relationship is further exemplified by varying the substituent at first position of the pyrazolopyridine ring **2**. However, the simple alkyl and aryl substituent on first position did not yield selective PDE4B inhibitors, except for the hydroxyethyl substituent in the pyrazolopyridine derivative **2e**, which exhibited a similar pIC_50_ of 6.8 as that of the lead molecule. Introduction of the amide function at fifth position retained PDE4B selectivity and secondary amides were well-tolerated because of their lipophilic nature, while benzyl amide derivative **3a** and saturated analogues such as **3b** showed enhanced inhibitory activity on PDE4B with pIC_50_ values of 8.5 and 8.1, respectively. Primary amide **3c** showed significant inhibition (pIC_50_ 6.2), but tertiary amide **3d** and cyclic amide **3e** exhibited low inhibitory potential towards PDE4B with pIC_50_ values of 7.3 and 6.8, respectively.

**Fig. 2.**
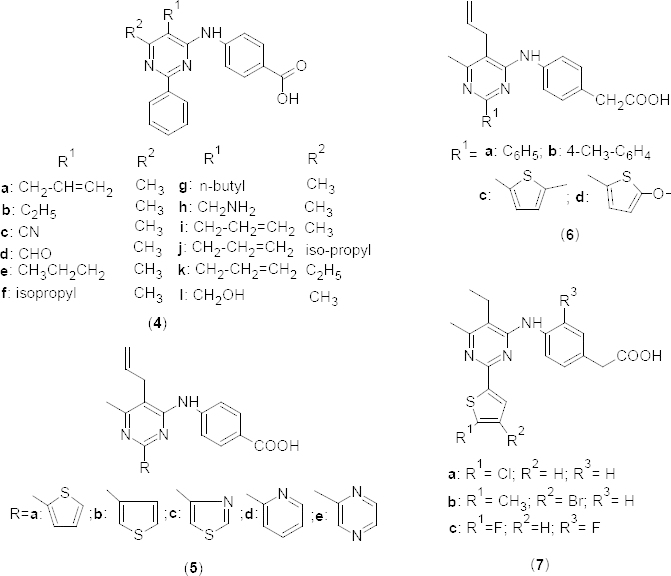
Analogues of 2-arylpyrimidines with PDE4B inhibitory activity

In the year 2009, 2-arylpyrimidine derivative **4a** (Figure 2) was identified as a selective and potent PDE4B inhibitor with an IC_50_ of sub-micromolar range (0.19 µM) and a potential 10-fold selectivity over PDE4D [38]. Authors further investigated the structure-activity relationship to optimize this lead compound which afforded a series of potent PDE4B inhibitors with >100-fold selectivity over the PDE4D isozyme. With a good pharmacokinetic profile, the selected compound exhibited potent anti-inflammatory properties. The compounds bearing allyl (**4a**), ethyl (**4b**), cyano (**4c**), or formyl (**4d**) groups at fifth position of the pyrimidine ring were found to be equipotent for PDE4B inhibition (pIC_50_190, 140, 120, and 300 nM, respectively) and 4B/4D selectivity. Bulkier groups at this position reduced the potency in derivatives **4f** (PDE4B pIC_50_ 1300 nM), **4g**, **4h**, and **4j** (PDE4B pIC_50_>1000 nM in all three compounds). Derivative **4k**, having an ethyl group at sixth position of the pyrimidine ring, showed enhanced potency (PDE4B pIC_50_ 34 nM), but less selectivity compared with the methyl analog **4a**. Authors also explored the SAR at second position of the pyrimidine ring. Potency and selectivity towards PDE4B was retained when the phenyl ring at second position of the pyrimidine ring was substituted with 2-thienyl **5a** (Figure 2) (PDE4B pIC_50_ 120 nM) or 3-thienyl **4b** (PDE4B pIC_50_ 68 nM) groups. However, substitution of thiazol-4-yl **5c** (PDE4B pIC_50_ 2800 nM), 2-pyridyl **5d** (PDE4B pIC_50_ 3700 nM), or pyradinyl **5e** (PDE4B pIC_50_ 860 nM) groups at the same position markedly reduced the potency. Similar results were observed when the phenyl ring was substituted at second position of the pyrimidine ring and a carboxymethyl group at fourth position of the aminophenyl moiety present at fourth position of the pyrimidine ring (**6a**) (Figure 2). However, compounds **6c** and **6d**, possessing a substituted thienyl group at R^1^ and a carboxymethyl group at second position of pyrimidine ring, showed more than a 25-fold selectivity towards PDE4B. Further modification of the above compounds resulted in compounds **7a–d** (Figure 2), which showed potent inhibitory activity on PDE4B (pIC_50_ 15 nM, 6.8 nM, and 15 nM, respectively) and >100-fold selectivity over PDE4D (PDE4B pIC_50_ 1700 nM, 2900 nM, 3100 nM, respectively).

**Fig. 3.**
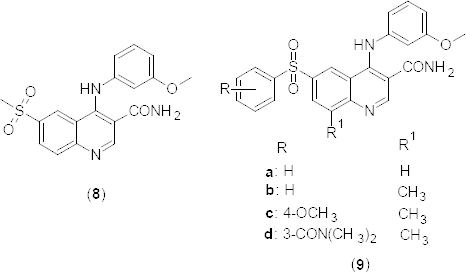
Analogues of 3-aminocarboxy-4-anilinoquinoline with PDE4B inhibitory activity

In another communication, the discovery of 3-aminocarboxy-4-anilino quinoline **8** (Figure 3) as a potent and selective PDE4 inhibitor (PDE4B pIC_50_ 8.4) is described [39]. Further structural modification of compound **8** has been carried out and the structure-activity relationship of a novel series of quinoline-3-carboxamides **9a–d** (Figure 3) at fourth, sixth, and 8-methyl compounds is described by the authors. A further compound, **9d** (GSK256066), was identified as a highly potent PDE4 inhibitor suitable for inhaled administration, which also inhibited the LPS-induced production of TNF-α from isolated human peripheral blood mononuclear cells with a pIC_50_ of 11.1, and negligible oral bioavailability in rats. The crystal structure of GSK256066 bound to PDE4B was also obtained to 1.75 Å resolution (PDB ID: 3GWT).

**Fig. 4.**
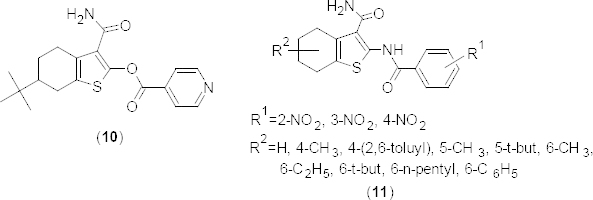
Structures of various congeners of 1-benzothiophene-3-carboxamides with PDE4B inhibitory activity

By high throughput screening, tetrahydrobenzothiophene (THBT) bisamide (**10**) (Figure 4) was discovered to be a potent and modest PDE4B over the 4D-selective inhibitor (PDE4B/4D pIC_50_ 6.7/6.5) [40]. Authors investigated the HBT binding mode with the ligand/PDE4B catalytic domain of the available co-crystal structure (PBD ID: 3HMV) [41] to design the analogue having PDE4B selectivity over PDE4D. Furthermore, based on the binding mode at the active site of PDE4B, disubstituted-4,5,6,7-tetrahydro-1-benzothiophene-3-carboxamides **11** (Figure 4) were synthesized and evaluated for their binding affinity to the PDE4B isoenzyme. Authors disclosed the structure-activity relationship at 3-carboxy and around the tetrahydrobenzo groups. Potencies significantly reduced when the primary amide was methylated and indicated a clear requirement for the primary carboxamide at third position. Structural modification around the tetrahydrobenzo group confirmed the 4B-subtype selectivity and indicated a preference for small C6 substituents. Moreover, the maximum potency and 10-fold selectivity was observed in the compound where R^2^ = 6-ethyl.

**Fig. 5.**
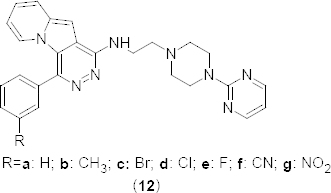
Analogues of pyridazino[4,5-*b*]indolizine with PDE4B inhibitory activity

**Fig. 6.**
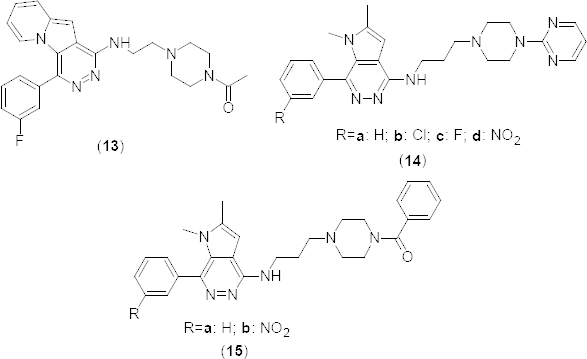
Analogues of 1,2-dimethyl-1*H*-pyrrolo[2,3-*d*]pyridazine with PDE4B inhibitory activity

In search of new compounds to treat inflammation, the pyridazino[4,5-*b*]indolizine analogue **12a** (Figure 5) was found to possess a 23-fold selectivity for PDE4B (K_i_ 2.60 lM) over PDE4D [42]. In order to improve potency and selectivity, the substituted phenyl analogues **12b–g** were prepared and comparable results were obtained from a cell-based adenylyl cyclase functional assay (PDE4B, IC_50_ 13.2 µM; PDE4D, IC_50_ >100 µM). Interestingly, the fluorine-substituted analogue **12e** was found to have high selectivity towards PDE4B compared to the derivative **12a**. In N-acetyl piperazine analogue **13** (Figure 6), the electron-withdrawing substituent on the 3-position of benzene significantly increased the PDE4B inhibitory potency (PDE4B K_i_ 1.90 µM). 1,2-Dimethyl-1*H*-pyrrolo[2,3-*d*]pyridazine analogues **14a–d** (Figure 6) were synthesized and evaluated for the selective affinity for PDE4B compared to PDE4D. Results revealed that -Cl and -Br substituents, respectively, at second and fourth position of the phenyl ring reduced the PDE4B affinity.

In continuation of the above work [42], two 1,2-dimethyl-1*H*-pyrrolo[2,3-*d*]pyridazine analogues, **15a** and **15b** (Figure 6) have been reported to possess greater affinity as well as selectivity for PDE4B. However, the analogue **15b** having the 3-NO_2_-C_6_H_5_ substituent on seventh position of the pyrrolo[2,3-*d*]pyridazine ring showed poor oral bioavailability in the pharmacokinetic study. On the other hand, unsubstituted analogue **15a** exhibited an impressive 205-fold selectivity for PDE4B when compared to PDE4D.

**Fig. 7.**
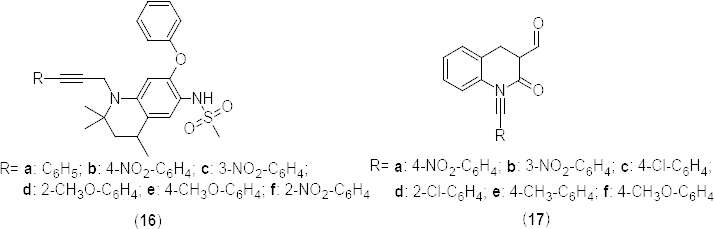
Analogues of 1-(arylmethylidyne)-1,2,3,4-tetrahydro-1λ^5^-quinoline with PDE4B inhibitory activity

In relation to the development of selective PDE4B inhibitors, a series of novel 1-(arylmethylidyne)-1,2,3,4-tetrahydro-1λ^5^-quinolines **16a–f** (Figure 7), structurally related, respectively, to nimesulide and their 2-oxo analogues and **17a–f** (Figure 7), have been designed and synthesized using Sonogashira coupling as a key step [43]. Synthesized compounds were evaluated *in vitro* for their PDE4B inhibitory activity at 30 µM. Compounds **16c** and **17d**, having 3-nitrophenyl and 2-chlorophenyl substituents, respectively, on the alkynyl chain attached to the nitrogen of the tetrahydroquinoline ring showed significant PDE4B inhibition (41.64 and 54.37%, respectively). Authors also presented *in silico* docking study results and interestingly, derivative **17c** showed both electrostatic and hydrophobic interactions with the PDE4B enzyme.

**Fig. 8.**
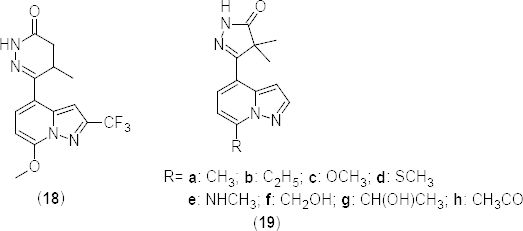
Analogues of pyrazolo[1,5-*a*]pyridine with PDE4B inhibitory activity

Further investigation established a basis for the development of potent PDE4-selective and dual PDE3/4-selective inhibitors [44]. In a separate communication [45] authors disclosed (−)-6-[7-methoxy-2-(trifluoromethyl)pyrazolo[1,5-a]pyridin-4-yl]-5-methyl-4,5-dihydro-3(2*H*)-pyridazinone (**18**) (Figure 8) as a dual PDE3/4 inhibitor (PDE4B IC_50_ 0.47) with potent bronchodilatory and anti-inflammatory activities and an improved therapeutic window over roflumilast in a number of *in vitro* and *in vivo* models used for pharmacological profiling. Based on the structure of compound **18** and in continuation of the above work, authors [46] disclosed a novel series of 4,4-dimethylpyrazolones **19a-h** (Figure 8) as dual PDE3/4 inhibitors. Potential bicyclic heteroaromatic replacement subunits for the pyrazolo[1,5-*a*]pyridine core of this series has also been undertaken and the activity of the resulting compounds is described. Synthesized compounds were evaluated to optimize the effect of substituents on seventh position of the pyrazolo[1,5-a]-pyridine nucleus. Compounds **19a-h** showed promising *in vitro* PDE4B inhibition (IC_50_ 0.16, 0.017 µM, 0.41 µM, 0.079 µM, 0.015 µM, 0.035 µM, 0.0034 µM, 0.0075 µM, respectively). Interestingly, the 7-ethyl derivative (**19b**) was found to be 25-fold more effective as a PDE4 inhibitor than the 7-methoxy analog (**19c**), revealing that strict catechol ether is not required and indeed potentially detrimental to PDE4-inhibitory activity.

**Fig. 9.**
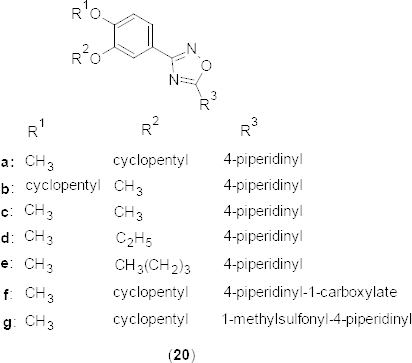
Analogues of 3,5-disubstituted-1,2,4-oxadiazole with promising PDE4B2 inhibitory activity

A novel series of 3,5-disubstituted-1,2,4-oxadiazoles **20a-g** (Figure 9) has been prepared and evaluated for PDE4B2 inhibitory activity [47]. Among the synthesized compounds, **20a** exhibited maximum PDE4B2 inhibition (IC_50_ 5.28 lM). A structure-activity relationship study revealed that the substituents 3-cyclopentyloxy-4-methoxyphenyl group at third position and the cyclic ring bearing heteroatoms at fifth position of the 1,2,4-oxadiazole ring are important for activity. The molecular modeling study showed similar interactions of the 3-cyclopentyloxy-4-methoxyphenyl group compared to the piclamilast. However, the heteroatom ring was found to be slightly deviated compared to piclamilast. Compound **20a** exhibited significant analgesic and anti-inflammatory activities in the formalin-induced pain in mice and carrageenan-induced paw edema model in rats.

**Fig. 10.**
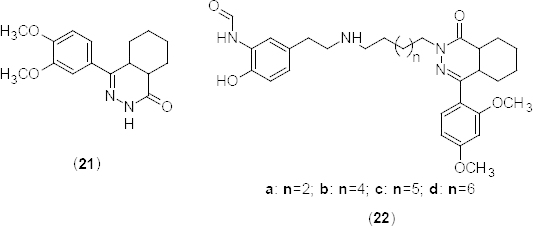
Analogues of hexahydrophthalazinone that have shown promising PDE4B2 inhibitory activity

Inspired by the development of multifunctional drugs, a new series of phthalazine analogues **21** and **22a–d** (Figure 10) is described [48] for the treatment of both asthma and COPD, which could target the β_2_-adrenoceptor and PDE4B_2_. Synthesized compounds **21** and **22a–d** exhibited PDE4B2 IC_50_ values of 0.520, 0.280, 0.278, 0.257, and 0.251 µM, respectively. Authors disclosed that the hexyl chain linking the hexahydrophthalazinone and *N*-[5-(2-aminoethyl)-2-hydroxyphenyl]formamide moieties (**22d**) is optimum for PDE4B2 inhibition. The synthesized compounds were also tested for guinea pig tracheal chain relaxation. Derivative (R)-(**22b**) showed significant relaxant effects on histamine-induced guinea pig tracheal chain contractions (pEC_50_ 9.3) compared to the reference drug isoprenaline (pEC_50_ 7.5).

**Fig. 11.**
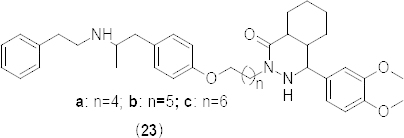
Formoterol–phthalazinone hybrids that have shown promising PDE4B2 inhibitory activity

In a similar work, PDE4B inhibitors bearing formoterol-phthalazinone hybrids were synthesized and evaluated as potent PDE4B2 inhibitors with β-agonist activity [49]. Three derivatives **23a-c** (Figure 11) exhibited potent PDE4B2 inhibition (IC_50_ 0.117 µM, 0.118 µM, and 0.104 µM, respectively). These compounds also displayed moderate–to-high β_2-_adrenoceptor agonist potency on isolated guinea pig tracheal rings pre-contracted with histamine. Compound (R,R)-**23c** showed the most potent agonist activity with a pEC_50_ value of approximately 9.0. Moreover, the same compound displayed strong PDE4B2 inhibitory activity with an IC_50_ of 0.092 µM.

**Fig. 12.**
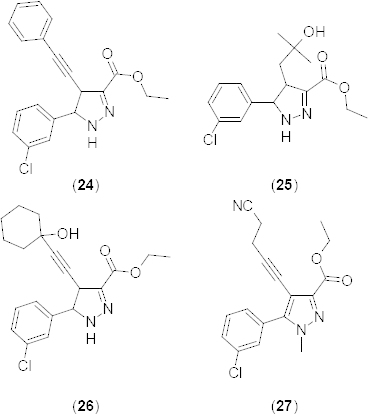
Analogues of ethyl 4,5-disubstituted-4,5-dihydropyrazole-3-carboxylate with PDE4B inhibitory activity

Ethyl 4,5-disubstituted-4,5-dihydropyrazole-3-carboxylates **24–27** (Figure 12) were synthesized through Pd/C–Cu catalysis as a new class of PDE4B inhibitors [50]. Synthesized compounds were evaluated against the PDE4B enzyme isolated from Sf9 cells and results were compared with the reference compound rolipram, a well-known inhibitor of PDE4. All compounds showed significant inhibition (>70%) of PDE4B when tested at 30 µM. A dose-response study was also carried out using compound **27** which showed dose-dependent inhibition of PDE4B. It is well-known that inhibition of the PDE4D subtype is linked to the emetic response [51]; hence, compounds **24-27** were tested for their PDE4B selectivity over PDE4D. Tested compounds showed 20–30% inhibition at 30 µM indicating their approximately two-fold selectivity towards PDE4B. The *in silico* docking study result of compound **25** showed the interaction of the hydroxyl function of Tyr233 with pyrazole nitrogen, the π–π stacking interaction between the pyrazole ring and His234, coordinate interaction between the Mg^+2^ ion and the ester carbonyl function, and coordinate interaction between the Zn^+2^ ion and pyrazole nitrogen. On the other hand, the π–π stacking interaction between pyrazole and His234 and the coordinate interaction between Mg^++^ and the ester carbonyl group was observed in compound **26**.

**Fig. 13.**
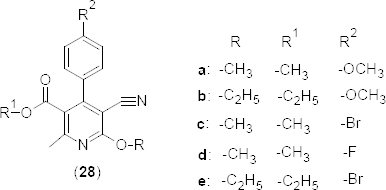
Cyano pyridine derivatives with PDE4B inhibitory activity

Green synthesis of functionalized cyano pyridines **28a–e** (Figure 13) as novel PDE4B inhibitors has been described via the montmorillonite K-10-mediated multi-component reaction in a chemo- and regioselective manner [52]. The synthesized compounds were evaluated for their PDE4B inhibitory potency at 30 µM using rolipram as the standard. The *in vitro* results revealed that a strong electron donating group such as OCH_3_ at the para position of the benzene ring in **28a** (percentage inhibition 64.10) and **28b** (percentage inhibition 65.0) was optimum for the activity compared to other milder electron donating groups such as F or Br (**28c–e**) (percentage inhibition <45). The di- or tri- substitution on the C-4 benzene ring resulted in the loss of PDE4B inhibitory activity. *In silico* docking studies showed the binding affinities of compounds **28a** and **28c** with a docking score of −25.47 and −17.8 kcal/mol, respectively. The cyano group of compound **28c** formed H-bonding with HIS234 of the PDE4B isoenzyme. However, it was the OCH_3_ group in **28a** that formed H-bonding with the Glu443 residue of PDE4B. In addition, it also showed an arene-arene interaction with the Phe446 residue.

**Fig. 14.**
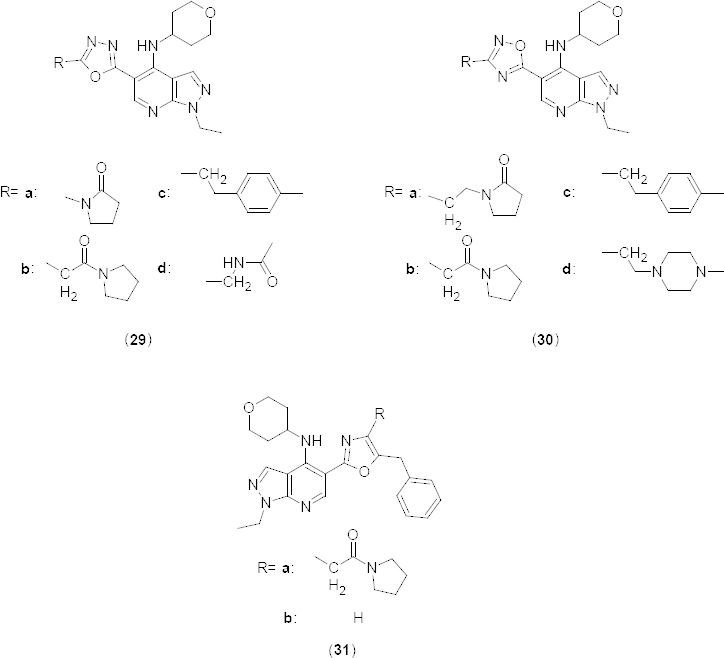
1,3,4-oxadiazole and 1,2,4-oxadiazole containing 1-ethyl-1*H*-pyrazolo[3,4-*b*]-pyridines with PDE4B inhibitory activity

1,3,4-oxadiazole and 1,2,4-oxadiazole containing 1-ethyl-1*H*-pyrazolo[3,4-*b*]pyridines **29a–d** and **30a–d** (Figure 14) have been explored as potent PDE4B inhibitors [39] (pIC_50_8.0–8.7 and 7.8–9.4, respectively) taking into account the discovery of 4-(substituted amino)-1-alkyl-pyrazolo[3,4-*b*]pyridine-5-carboxamides as potent and selective PDE4B inhibitors [37]. The structure-activity relationship of fifth 5-position was studied [53] further. Preliminary structure–activity studies were conducted against isolated PDE4B, the predominant PDE4 subtype in inflammatory cells of interest. Results indicated that both oxadiazole isomers were tolerated at fifth position. Different oxadiazole substituents showed significant potency in each series, but unfortunately no structure-activity relationship was established. Further di-substituted and mono-substituted oxazoles **31a** and **31b** (Figure 14) were also synthesized. The di-substituted oxazole **31a** demonstrated sub-nM activity in the isolated enzyme and isolated human peripheral blood mononuclear cells (PBMC) assay and gave at least a 10-fold increase in potency over 1,3,4-oxadiazole **13c** and 1,2,4-oxadiazole **29b**. Moreover, in compound **31a**, the introduction of a second substituent showed an increase in potency in comparison to the mono-substituted oxazole **31b**.

**Fig. 15.**
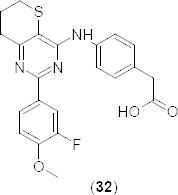
Structure of 7,8-dihydro-6*H*-thiopyrano[3,2-*d*]pyrimidine analogue, an orally active PDE4B-selective inhibitor

In the year 2013, compound **32** (Figure 15) was identified as an orally active PDE4B-selective inhibitor over PDE4D both in humans (80-fold selective) and mice (29-fold selective) [54]. The therapeutic effect of compound **32** was evaluated on lipopolysaccaride (LPS) injection–induced plasma TNF-α elevation and on LPS inhalation-induced pulmonary neutrophilia in mice. The therapeutic index for TNF-α production (TI^TNF^ = ID_50_ in gastric emptying/ID_50_ in LPS injection-induced plasma TNF-α elevation) of compound **32** was larger than roflumilast (9.0 and 0.2, respectively), whereas the therapeutic index for pulmonary neutrophilia of compound **32** was comparable to roflumilast (1.0 and 0.5, respectively). Authors disclosed that the TI^TNF^ of compound **32** was not superior compared to that of roflumilast in spite of its high selectivity for PDE4B over PDE4D in mice.

**Fig. 16.**
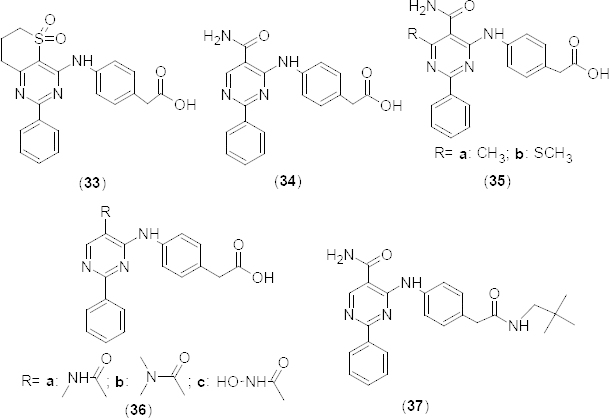
Structure of 2-phenylpyrimidine analogues showing selective PDE4B inhibitory activity

In a separate communication, 2-phenylpyrimidine **33** and 5-carbamoyl-2-phenylpyrimidine **34** derivatives (Figure 16) were reported as potent PDE4B (IC_50_ 25 nM and 200 nM, respectively) and mTNF-α (IC_50_ 390 and 690 nM, respectively) inhibitors [55]. Compounds were also evaluated *in vivo* against LPS-induced pulmonary neutrophilia in mice. In compound **34**, substitution of methyl (**35a**) (PDE4B IC_50_ 4000 nM) or methylthio (**35b**) (PDE4B IC_50_ 2000 nM) groups (Figure 16) on sixth position of the pyrimidine ring markedly reduced the PDE4B inhibitory activity. Moreover, substitution of N-methylacetamide (**36a**) (PDE4B IC_50_ 8200 nM), N,N-dimethylacetamide (**36b**) (PDE4B IC_50_ 8% at 10 µM), and N-hydroxyacetamide (**36c**) (PDE4B IC_50_ 42% at 3 µM) groups (Figure 16) on fifth position of the pyrimidine ring resulted in a marked decrease in potency, whereas derivative **37** (Figure 16) with the *N*-(2,2-dimethylpropyl)acetamide substituent on fourth position of the phenyl ring exhibited maximum *in vitro* inhibitory activities against PDE4B (IC_50_ 8.3 nM) and TNF-α (IC_50_ 3.0 nM). The 5-carbamoyl moiety of compound **37** exhibited a water-bridged hydrogen bonding network with Asn395 and Gln443 residues in an X-ray crystallography study. In derivatives **36a–c**, substituents hindered these interactions and this may be the reason for the lowered potency. The higher potency of **37** is further supported by the lengthy neopentyl substituent which fits into the lipophilic pocket.

**Fig. 17.**
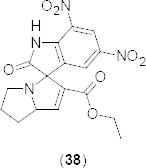
Structure of spirooxindole that showed promising PDE4B inhibitory activity

A novel series of spirooxindoles have been synthesized in the one-pot efficient methodology using Cu-mediated 1,3-dipolar cycloaddition of azomethineylides with dipolarophiles and evaluated for *in vitro* inhibition of PDE4B [56] using rolipram as a reference compound. The structure-activity relationship study revealed compound **38** (Figure 17) as a potent PDE4B inhibitor with >40% inhibition at 30 µM. In the *in silico* docking result, compound **38** C=O function showed two hydrogens bonding with His278 and Met347 residues of the active site. Additionally, hydrophobic interactions with the hydrophobic clamp residue of the Q-pocket were also observed.

**Fig. 18.**
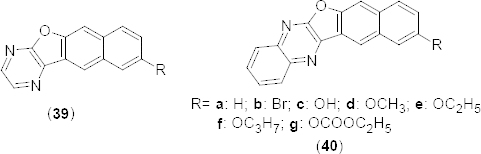
Structure of benzofuran analogues that showed promising PDE4B inhibitory activity

One-pot synthesis of benzofuran fused N-heterocycles **39** and **40a–g** (Figure 18) has been achieved by AlCl_3_-mediated C–C followed by C–O bond formation between 2,3-dichloropyrazine or its derivatives and phenols. Synthesized compounds were tested *in vitro* for their PDE4B inhibitory activity [57]. Compound **39** showed significant PDE4B inhibition at 30 µM in comparison to the reference compound rolipram. Compounds possessing a substituent on the benzofuran moiety showed moderate activities and the presence of a smaller substituent was well-tolerated compared to a larger or bulky group in terms of PDE4 inhibition.

**Fig. 19.**
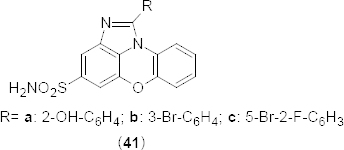
Novel series of imidazophenoxazine-4-sulfonamides that have shown promising PDE4B inhibitory activity

A number of novel imidazophenoxazine-4-sulfonamides **41a-c** (Figure 19) have been designed as potential inhibitors of PDE4. Designed compounds were synthesized in a multi-step reaction process involving the construction of a 1-nitro-10H-phenoxazine ring and then fusion with an imidazole ring as the key steps [58]. Some of these compounds showed promising *in vitro* PDE4B and D inhibition when tested. Compounds bearing 2-hydroxyphenyl (**41a)**, 2-bromophenyl (**41b)**, and 5-bromo-2-fluorophenyl (**41c**) exhibited dose-dependent inhibition of PDE4B with IC_50_ values of 3.31 ± 0.62, 1.23 ± 0.18, and 0.53 ± 0.18 µM, respectively. The interaction of compound **41a** with the PDE4B was mainly contributed by H-bonding between the amino group of **41a** with His-278,H-bonding between the hydroxyl group of **41a** and Asp392, and two π–π stacking interactions between the benzimidazole moiety and His234. Similarly, the interaction of compound **41b** with the PDE4B was contributed by H-bonding between the NH_2_-group of **41b** and Thr345, as well as Asp392, π–π stacking interactions between the central 1,4-oxazine ring and Tyr233, and π–π stacking interactions between the phenyl group of **41b** and Phe446. In compound **41c**, H-bonding between the amino group and Asp275 and π–π stacking interactions between the aromatic ring system and Tyr233, His234, and Phe446 residues of PDE4B was observed.

**Fig. 20.**
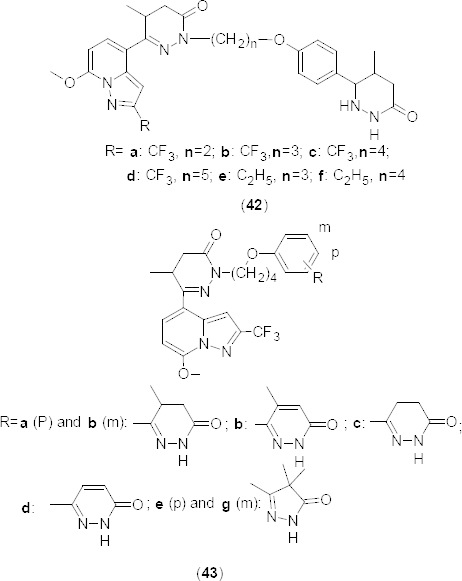
Novel series of N-alkylated pyridazinones that have shown selective PDE4B inhibitory activity

Yet in another publication [59], authors explored the effect of N-alkylation of the pyridazinone ring in compound **18** (Figure 8) on PDE4B inhibition and selectivity. N-alkylation of the pyridazinone ring resulted in a marked enhancement of potency against PDE4, but suppressed PDE3 inhibition. Addition of a 6-aryl-4,5-dihydropyridazin-3(2*H*)-one extension to the N-alkyl group in derivatives **42a–f** (Figure 20) facilitated both the enhancement of PDE4-inhibitory activity and the restoration of potent PDE3 inhibition. These modifications of structural changes afforded potent dual PDE3/4 inhibitors and most of the synthesized compound suppressed histamine-induced bronchoconstriction *in vivo* and exhibited promising anti-inflammatory activity *via* intratracheal administration.

The selectivity towards PDE4B increased by 23- to 350-fold in the trifluoromethyl substituted series **42a–d** (IC_50_ 0.66, 3, 0.3, 0.2 nM, respectively) compared to the lead compound **18** (PDE4B IC_50_ 70 nM), whereas the ethyl substituted derivatives **42e** and **42f** exhibited a lower PDE4B selectivity than its congeners (IC_50_ 5.9 and 1.7 nM, respectively). Considering the variations in selectivity of compounds possessing tetramethylene (**42c**) and pentamethylene (**42d**), linkers were further explored for the structure-activity relationship with 5-methyl-3,4-dihydropyridazinone and 4,4-dimethylpyrazolone subunits (**43a–g**) (Figure 20). *In vitro* screening results revealed a reduced PDE3A selectivity with moderate inhibition of PDE4B in compounds **43b** and **43d** (PDE4B IC_50_ 6 nM and 20 nM, respectively) having a fully unsaturated pyridazanone nucleus [59]. Varying the 5-methyl-3,4-dihydropyridazinone ring from fourth position in **43a** (PDE4B IC_50_ 0.3 nM) to third position in **43b** (PDE4B IC_50_ 0.5 nM) retained the selectivity for PDE4B at the cost of PDE43A selectivity. Based upon the *in vitro* results, compounds having 4,5-dihydropyridazin-3(2*H*)-one (**43c**, PDE4B IC_50_ 0.1 nM) and 4,4-dimethyl-1*H*-pyrazol-5(4*H*)-one (**43f**, PDE4B IC_50_ 0.5 nM) rings were found to be promising in PDE4B inhibition.

**Fig. 21.**
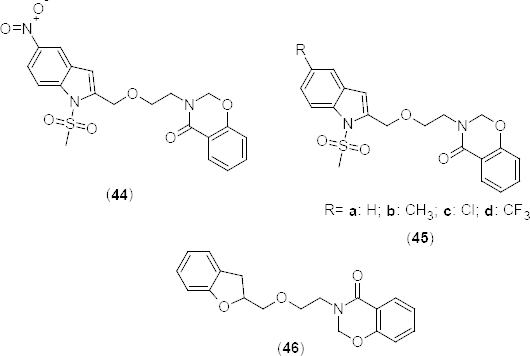
Structure of benzoxazinone derivatives that have shown potent PDE4B inhibitory activity

Compound **44** (Figure 21) was discovered as a virtual hit in a docking study where the benzoxazinone moiety carbonyl function formed a H-bond with His234 of the metal binding pocket and the N-methanesulfonyl oxygen formed a H-bond with Gln443 of the Q pocket in the active site. These interactions were similar to that of the known inhibitor rolipram [60]. 2*H*-1,3-benzoxazin-4(3*H*)-one derivatives **45a-d** and **46** (Figure 21) containing respectively 1-(methylsulfonyl)-1*H*-indole or benzofuran moieties were synthesized by Pd/C–Cu-mediated coupling-cyclization. The o-iodoanilides or o-iodophenol were coupled with 3-{2-(prop-2-ynyloxy)ethyl}-2*H*-benzo[*e*][1,3]oxazin-4(3*H*)-one using 10% Pd/C–CuI–PPh3 as the catalyst and triethylamine as the base. Synthesized compounds were tested *in vitro* for their PDE4B inhibitory potential using a cell-based cAMP reporter assay. The tested compounds exhibited potent inhibition of PDE4B at 30 µM. Compound **44** showed a fivefold increase over the control, whereas rolipram showed a nine-fold increase. In the dose-response curve, compound **44** also showed a dose-dependent increase in cAMP levels.

**Fig. 22.**
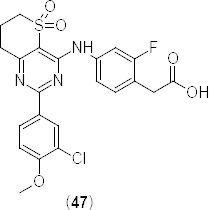
Structure of the 5,5-dioxo-7,8-dihydro-6*H*-thiopyrano[3,2-*d*]pyrimidine analogue that has shown highly selective PDE4B inhibition

In search of highly selective PDE4B inhibitors, a novel series of 5,5-dioxo-7,8-dihydro-6*H-*thiopyrano[3,2-*d*]pyrimidines were synthesized and evaluated for their PDE4B subtype selectivity using human PDE4B2 and PDE4D2 full-length enzymes [61]. The molecules were optimized by varying the substituents on the pyrimidine ring and the phenyl ring present over the side chain. The *in vitro* screening of the synthesized compounds resulted in the identification of 2-(3-chloro-4-methoxyphenyl)-5,5-dioxo-7,8-dihydro-6*H*-thiopyrano[3,2-*d*]pyrimidine (**47)** (Figure 22) as a highly selective PDE4B inhibitor, which showed PDE4B inhibitory activity with an IC_50_ value of 3.0 nM and 433-fold PDE4B selectivity over PDE4D.

Selective PDE4 inhibitors exhibit antiproliferative activity against T-cells and B-cells and this finding helped researchers to investigate the selective PDE4 inhibitors as novel anticancer agents. This was further supported by the antiproliferative activity of PDE4 inhibitors against murine carcinoma cells [62]. PDE4 inhibitor rolipram induced cell cycle arrest and apoptosis [63], and overexpression of PDE4B resulted in diffuse large cell lymphoma (DLBCL); a fatal malignancy indicating that PDE4B inhibitors could be useful anticancer agents [64]. Moreover, PDE4B selective inhibitors were reported for selective apoptosis in malignant cells without affecting the normal cells. Based on these facts, novel PDE4B inhibitors were developed and screened for anticancer activity [65].

**Fig. 23.**
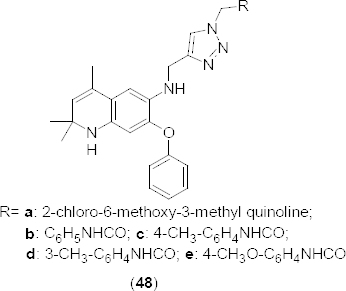
Structure of 1,2-dihydroquinolinemethanesulfonamide analogues that have shown potent PDE4B inhibition and anticancer activity

In the year 2013, novel *N*-[(1-substituted-1*H*-1,2,3-triazol-4-yl)methyl]-*N*-2,2,4-trimethyl-7-phenoxy-1,2-dihydroquinolinemethanesulfonamides **48a–e** (Figure 23) were synthesized as selective PDE4B inhibitors. The reaction was carried out in aqueous DMF *via* a multi-step sequence consisting of copper-catalyzed azide-alkyne cycloaddition (CuAAC) as the key step [66]. The compounds were evaluated *in vitro* for their cytotoxic activity using the lung adenocarcinoma epithelial cell line (A549), prostate cancer cell line (DU145), cervical cancer cell line (HeLa), and hepatocellular liver carcinoma cell line (HepG2) in an MTT assay. Among the synthesized inhibitors, **48a** and **48b** showed promising activity against the human lung cancer cell line (A549) with an IC_50_ of 8–9 µM. Although compounds **48b**, **48d**, and **48e** were identified as potent PDE4B inhibitors, they showed no cytotoxic activity towards the tested cancer cell lines. The docking results of the synthesized compounds revealed a high binding affinity of compound **48d** with 1XMY (PBD ID). Hydrogen bonding with HIE-234 and π-π stacking interactions with PHE446 residues were also observed.

**Fig. 24.**
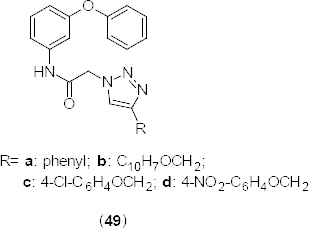
Structure of 1,2,4-triazole analogues that have PDE4B inhibition and anticancer activity

The novel nimesulide-based new class of triazole derivatives **49a–d** (Figure 24) were synthesized as PDE4B inhibitors [67]. The synthesized compounds were tested against cancer cells, keeping in view the reported anticancer activity of nimesulide [68] and the anti-inflammatory activity of 1,2,4-triazoles [69]. Some of the synthesized compounds exhibited significant *in vitro* PDE4B inhibitory properties with >50% inhibition at 30 µM. Moreover, results were also supported by the docking studies. The *in vitro* anticancer activity was evaluated using the HCT-15 human colon cancer cell lines with doxorubicin [IC_50_ 50 µg/mL, (0.09 µM)], an anthracyclin antibiotic as the reference compound. Two of the synthesized compounds **49c** and **49d** exhibited IC_50_ values in the range 21-22 µg/mL. Docking results of synthesized compounds revealed similar interactions with Gln443 as that of rolipram. Apart from Gln443, compound **49a** also exhibited interactions with His234 and His278, whereas compound **49b** and **49c** showed binding affinity for Asn283.

During cardiac action potential, Ca^2+^ influx through the sarcolemma L-type Ca^2+^ channels (LTCC) triggers Ca^2+^ release from ryanodine receptor 2 located in the sarcoplasmic reticulum; this results in elevated Ca^2+^ throughout the cell leading to contraction of the myocardium. PDE4 is one of the main PDEs expressed in the heart. PDE4 becomes predominantly active upon β-adrenergic receptor stimulation and regulates LTCC and cAMP levels in cardiomyocytes [70–75]. In PDE4B-deficient mice, increased expression of the β-adrenoreceptor along with LTCC was observed leading to elevated intracellular Ca^2+^ and cell contraction [76].

**Fig. 25.**
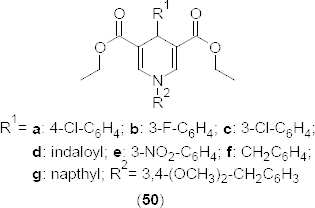
Structure of 1,4-dihydropyridine analogues that have Ca^2+^ channel blocking and

In another study, a novel series of 1,4-dihydropyridine-based PDE4B inhibitors were synthesized [77] and evaluated for their *in vitro* Ca^2+^ channel blocking and PDE4B inhibitory activities. Compounds **50a-g** (Figure 25) exhibited >80% inhibition of PDE4B. Among the synthesized inhibitors, **50d**, having an indaloyl moiety, exhibited significant selectivity towards PDE4B with a docking score of −8.1 kcal*/*mol. The *in silico* docking result of compound **50d** supported the reason for the selectivity, where the indole moiety exists in two different orientations in PDE4D, and in both cases, it makes a stronger hydrogen bond with Glu396 or Glu505 residues of PDE4D, while its orientation in PDE4B is completely different than that in PDE4D. The dimethoxy group of **50d** interacted with the metal atoms in PDE4B, while the indole moiety was shown to be H-bonded with the Ser442 backbone.

In recent research [78], authors disclosed that PDE4B selectivity can be achieved by the capture of a C-terminal regulatory helix, known as CR3 (control region 3), across the active site in a conformation that closes access by cAMP. PDE4B selectivity is driven by a single amino acid polymorphism in CR3 (Leu674 in PDE4B1 versus Gln594 in PDE4D). A new co-crystal structure with a bound ligand was developed which provided a guide map for the design of PDE4B-selective anti-inflammatory drugs. The comparative binding modes of the 2-arylpyrimidine derivative with PDE4D (PDB: 3G58), NVW-PDE4B (PDB: 3W5E), and OCP-PDE4B (PDB: 3KKT) structures showed that small molecules can interact with different residues of the CR3 helix resulting in multiple closed conformations. The study highlighted the significance of (PDE4B Leu674/PDE4DGln594 and PDE4B Met675/PDE4D Thr595) that is essential to engage the ligand and/or catalytic domain.

## Conclusion

Advancements in PDE4B selective-targeted therapies have shown promise in recent years for the treatment of inflammation, chronic obstructive pulmonary disorders, cancers, and myocardium contractility disorder. After the development of the prototypic PDE4 inhibitor rolipram, more selective inhibitors targeting the PDE4B isozyme have been widely investigated. However, the biological activity of this class of compounds deserves further investigation. This is evident when inflammatory diseases are considered. Although the research on this subject is incipient, the number of reports disclosing the effects of PDE4B inhibitors on TNF-α production and Ca^2+^ channel blockage has recently been increasing. PDE4B selective inhibitors have been shown to be promising, which calls for the design of more efficient anti-inflammatory, anticancer, and cardio-protective agents. Although some patterns appear relating the structure and the pharmacological field, this also should be considered when designing new selective PDE4B inhibitors.

## Abbreviations

PDE: phosphodiesterases;
cAMP: cyclic adenosine monophosphate;
cGMP: cyclic guanosine monophosphate;
pIC_50_: negative logarithm of the half maximal inhibitory concentration;
COPD: chronic obstructive pulmonary disease;
TNF: tumor necrosis factor;
nM: nanomol;
PDB: protein data bank;
IC_50_: half maximal inhibitory concentration;
pEC_50_: negative logarithm of half maximal effective concentration;
µM: micromol;
LPS: lipopolysaccharide;
MTT: 3*-*(4,5-dimethylthiazol-2-yl)*-*2,5-diphenyltetrazolium bromide;
LTCC: sarcolemma L-type Ca^2+^ channels;o
SAR: structure-activity relationship;
CR3: control region 3;
NVW: *N*-*tert*-butyl-2-{4-[(5,5-dioxido-2-phenyl-7,8-dihydro-6*H*-thiopyrano[3,2-*d*]-pyrimidin-4-yl)amino]phenyl}acetamide;
OCP: 5-{3-[(1S,2S,4R)-bicyclo[2.2.1]hept-2-yloxy]-4-methoxyphenyl}-tetrahydropyrimidin-2(1H)-one;
